# Supplementary material for: Genetic signature of blind reintroductions of Iberian ibex (Capra pyrenaica) in Catalonia, Northeast Spain
Source: PLoS One. 2022 Sep 21;17(9):e0269873. doi: 10.1371/journal.pone.0269873 (PMC9491545; doi:10.1371/journal.pone.0269873)
Supplement: S1 File — (DOCX) [file pone.0269873.s001.docx]

**Supporting Material 1.** **Microsatellite markers used in the study, with reference, Multiplex, GenBank Accession number (GB acc.numb.) and Annealing Temperature (Ta).**

| **Marker** | **Reference** | **Multiplex** | **GB acc. numb.** | **Ta (^○^C)** |
| --- | --- | --- | --- | --- |
| ILSTS29 | Kemp et al., 1995; Angelone-Alasaad et al., 2017 | I | L37252 | 59 |
| MILSTS076 | Kemp et al., 1995; Angelone-Alasaad et al., 2017 | I | 9982 | 59 |
| OARFCB193 | Buchanan et al., 1994; Angelone-Alasaad et al., 2017 | I | L01533 | 59 |
| ETH10 | Toldo et al., 1993; Angelone-Alasaad et al., 2017 | II | Z22739 | 55 |
| MAF36 | Swarbrick et al., 1991; Angelone-Alasaad et al., 2017 | II | M80519 | 55 |
| OarKP6 | Paterson & Crawford, 2000; Angelone-Alasaad et al., 2017 | II | AF223411 | 55 |
| BM4505 | Bishop et al., 1994; Angelone-Alasaad et al., 2017 | II | G18511 | 55 |
| BM1258 | Bishop et al., 1994; Angelone-Alasaad et al., 2017 | II | G18385 | 55 |
| SR-CRSP-8 | Ma et al., 1996; Angelone-Alasaad et al., 2017 | III | U21788 | 55 |
| BM1818 | Bishop et al., 1994; Angelone-Alasaad et al., 2017 | III | G18391 | 55 |
| URB058 | Bishop et al., 1994; Angelone-Alasaad et al., 2017 | III | U21788 | 55 |
| BM1225 | Bishop et al., 1994; Angelone-Alasaad et al., 2017 | IV | G18419 | 59 |
| IDVGA30 | Mezzelani et al., 1995; Angelone-Alasaad et al., 2017 | IV | X85049 | 59 |
| JMP29 | Lumsden et al., 1996; Angelone-Alasaad et al., 2017 | IV | U30893 | 59 |
