# Supplementary material for: Genetic signature of blind reintroductions of Iberian ibex (Capra pyrenaica) in Catalonia, Northeast Spain
Source: PLoS One. 2022 Sep 21;17(9):e0269873. doi: 10.1371/journal.pone.0269873 (PMC9491545; doi:10.1371/journal.pone.0269873)
Supplement: S2 File — (DOCX) [file pone.0269873.s002.docx]

**Supporting Material S2. GeneBank accession numbers (GB acc. numb.) for the *cyt b* sequences from domestic goat (*C. hircus*), Alpine ibex (*C. ibex*) and Iberian ibex (*C. pyrenaica*) used in both the haplotype network and phylogenetic tree.**

| **Species** | **GB acc. numb.** | **References** |
| --- | --- | --- |
| *C. hircus* | AB004070.1-75.1 | Takada et al., 1997 |
|  | AB044307.1, AB044308.1 | Mannen et al., 2001 |
|  | AB110594.1, AB110595.1, AB110596.1, AB110597.1 | Sultana et al., 2003 |
|  | AB736095.1, AB736106.1, AB736119.1, AB736131.1, AB736144.1 | Nomura et al., 2013 |
|  | AF217254.1 | Kahila Bar-Gal, 2000 |
|  | D84201.1 | Arai et al., 1997 |
|  | DQ073048.1, DQ093614.1 | Das & Ghosh, 2005* |
|  | DQ089474.1-80.1 | Chen et al., 2005 |
|  | DQ514544.1-48.1 | Pidancier et al., 2006 |
|  | EU130773.1-80.1 | Liu et al., 2009 |
|  | EU259119.1-32.1 | An et al., 2007* |
|  | EU350118.1-33.1 | Huang et al., 2007* |
|  | FJ556557.1, FJ556564.1 | Lestari et al., 2018 |
|  | GQ141259.1-65.1 | Wu & Liu, 2009* |
|  | JX010743.1-46.1 | Kim et al., 2012* |
|  | KT283248.1, KT283253.1 | Reznik et al., 2015* |
|  | KT750046.1 | Amer & Al-Harbi, 2016 |
|  | KU253480.1 | Carnieli et al., 2016 |
|  | KX980488.1-90.1 | Hussain et al., 2016* |
|  | KY348291.1-330.1 | Gao et al., 2018 |
|  | KY366506.1, KY662383.1 | Benderskyi et al., 2016* |
|  | MF101245.1-58.1 | Kivila et al., 2018 |
|  | MF871361.1-517.1 | Deng, 2017* |
|  | MG923212.1-315.1 | Kamalakkannan et al., 2018 |
|  | MK159198.1-237.1 | De et al., 2018* |
|  | X56289.1 | Irwin et al., 1991 |
|  | AF034735.1 | Hassanin et al., 1998 |
|  | AF217256.1 | Kahila Bar-Gal & Greenblatt, 1999* |
| *C. ibex* | AJ010055.1 | Manceau, Crampe, et al., 1999 |
|  | AJ009879.1 | Manceau, Després, et al., 1999 |
|  | DQ514550.1-52.1 | Pidancier et al., 2006 |
|  | KT246295.1 | Benderskyi et al., 2015* |
|  | MF139798.1, MF139808.1, MF139817.1, MF139822.1 | Ureña et al., 2018 |
| *C. pyrenaica* | AJ010047.1-56.1 | Manceau, Crampe, et al., 1999 |
|  | EU081020.1-41.1 | Márquez et al., 2020 |
|  | MF139799.1, MF139800.1, MF139801.1, MF139803.1, MF139804.1, MF139805.1, MF139806.1, MF139809.1, MF139810.1, MF139811.1, MF139814.1, MF139816.1, MF139818.1, MF139819.1, MF139824.1, MF139825.1 | Ureña et al., 2018 |
|  | MG573148.1-50.1 | Ruiz et al., 2017* |
|  | H1 – to be submitted no Genbank | This study |
|  | H2 – to be submitted no Genbank | This study |
|  | H3 – to be submitted no Genbank | This study |
|  | H4 – to be submitted no Genbank | This study |
|  | H5 – to be submitted no Genbank | This study |
|  | H6 – to be submitted no Genbank | This study |
|  | H7 – to be submitted no Genbank | This study |
|  | H8 – to be submitted no Genbank | This study |
|  | H9 – to be submitted no Genbank | This study |
|  | H11 – to be submitted no Genbank | This study |
|  | H13 – to be submitted no Genbank | This study |
|  | H14 – to be submitted no Genbank | This study |

^*^ unpublished

Amer, S. A. M., & Al-Harbi, M. S. (2016). Amplification and sequencing of energy related mitochondrial genes for some domestic animals in Saudi Arabia. Journal of Camel Practice and Research, 23(1), 19–23. https://doi.org/10.5958/2277-8934.2016.00003.5

Angelone-Alasaad, S., Biebach, I., Pérez, J. M., Soriguer, R. C., & Granados, J. E. (2017). Molecular Analyses Reveal Unexpected Genetic Structure in Iberian Ibex Populations. PLOS ONE, 12(1), e0170827. https://doi.org/10.1371/journal.pone.0170827

Arai, K., Munechika, I., Ito, I., Kikkawa, A., Nakamura, K., Kanazawa, T., & Kosugiyama, M. (1997). Phylogenetic Relationship of Caprini Estimated by Cytochrome b Gene Sequence Analysis. Nihon Chikusan Gakkaiho, 68(2), 148–155. https://doi.org/10.2508/chikusan.68.148

Bishop, M. D., Kappes, S. M., Keele, J. W., Stone, R. T., Sunden, S. L. F., Hawkins, G. A., Toldo, S. S., Fries, R., Grosz, M. D., & Yoo, J. (1994). A genetic linkage map for cattle. Genetics, 136(2), 619–639. https://doi.org/10.1093/genetics/136.2.619

Buchanan, F. C., Galloway, S. M., & Crawford, A. M. (1994). Ovine microsatellites at the OarFCB5, OarFCB19, OarFCB20, OarFCB48, OarFCB129 and OarFCB226 loci. Animal Genetics, 25(1), 60–60. https://doi.org/10.1111/j.1365-2052.1994.tb00069.x

Carnieli, P., Castilho, J. G., Oliveira, R. de N., Brandão, P. E., & Batista, H. B. de C. R. (2016). Identification of different species of mammalians involved in zoonoses as reservoirs or hosts by sequencing of the mitochondrial DNA cytochrome B gene. Annual Research and Review in Biology, 10(1), 1–8. https://doi.org/10.9734/ARRB/2016/25230

Chen, S. Y., Su, Y. H., Wu, S. F., Sha, T., & Zhang, Y. P. (2005). Mitochondrial diversity and phylogeographic structure of Chinese domestic goats. Molecular Phylogenetics and Evolution, 37(3), 804–814. https://doi.org/10.1016/j.ympev.2005.06.014

Gao, J., Lyu, Y., Dai, J., Zhang, D., Yi, J., Li, H., Liu, C., & Sun, F. (2018). Phylogenetic research on Chongming Island’s white goats based on mitochondrial cytochrome b genes. Acta Agriculturae Shanghai, 34(1), 74–78.

Hassanin, A., Pasquet, E., & Vigne, J. D. (1998). Molecular systematics of the subfamily Caprinae (artiodactyla, bovidae) as determined from cytochrome b sequences. Journal of Mammalian Evolution, 5(3), 217–236. https://doi.org/10.1023/A:1020560412929

Irwin, D. M., Kocher, T. D., & Wilson, A. C. (1991). Evolution of the cytochrome b gene of mammals. Journal of Molecular Evolution, 32(2), 128–144. https://doi.org/10.1007/BF02515385

Kahila Bar-Gal, G. (2000). Genetic changes in Capra species of Southern Levant over the past 10,000 years as studied by DNA analysis of ancient and modern populations. The Hebrew University of Jerusalem.

Kamalakkannan, R., Jose, J., Thomas, S., Prabhu, V. R., & Nagarajan, M. (2018). Genetic diversity and maternal lineages of south Indian goats. Molecular Biology Reports, 45(6), 2741–2748. https://doi.org/10.1007/s11033-018-4322-5

Kemp, S. J., Hishida, O., Wambugu, J., Rink, A., Teale, A. J., Longeri, M. L., Ma, R. Z., Da, Y., Lewin, H. A., Barendse, W., & Teale, A. J. (1995). A panel of polymorphic bovine, ovine and caprine microsatellite markers. Animal Genetics, 26(5), 299–306. https://doi.org/10.1111/j.1365-2052.1995.tb02663.x

Kivila, J. N., Githui, E. K., Amugune, N. O., Githaiga, J. M., & Okwany, Z. A. (2018). Mitochondrial DNA Indicate that Kenyan Indigenous Goat (Capra hircus) Population is Diverse. Agricultural Research & Technology: Open Access Journal, 18(1), 1–8. https://doi.org/10.19080/ARTOAJ.2018.18.556044

Lestari, D. A., Purbowati, E., Sutopo, S., & Kurnianto, E. (2018). Amino acid sequence based on Cytochrome b gene in Kejobong goat and its genetic relationships among several local goats in Asia. Veterinary World, 11(8), 1196–1202. https://doi.org/10.14202/vetworld.2018.1196-1202

Liu, Y. P., Cao, S. X., Chen, S. Y., Yao, Y. G., & Liu, T. Z. (2009). Genetic diversity of Chinese domestic goat based on the mitochondrial DNA sequence variation. Journal of Animal Breeding and Genetics, 126(1), 80–89. https://doi.org/10.1111/j.1439-0388.2008.00737.x

Lumsden, J. M., Lord, E. A., & Montgomery, G. W. (1996). Characterization and linkage mapping of ten sheep microsatellite markers derived from a sheep x hamster cell hybrid. Animal Genetics, 27(3), 203–206. https://doi.org/10.1111/j.1365-2052.1996.tb00953.x

Ma, R. Z., Russ, I., Park, C., Heyen, D. W., Beever, J. E., Green, C. A., & Lewin, H. A. (1996). Isolation and characterization of 45 polymorphic microsatellites from the bovine genome. Animal Genetics, 27(1), 43–47. https://doi.org/10.1111/j.1365-2052.1996.tb01175.x

Manceau, V., Crampe, J.-P., Boursot, P., & Taberlet, P. (1999). Identification of evolutionary significant units in the Spanish wild goat, Capra pyrenaica (Mammalia, Artiodactyla). Animal Conservation, 2(1), S1367943099000335. https://doi.org/10.1017/S1367943099000335

Manceau, V., Després, L., Bouvet, J., & Taberlet, P. (1999). Systematics of the Genus Capra Inferred from Mitochondrial DNA Sequence Data. Molecular Phylogenetics and Evolution, 13(3), 504–510. https://doi.org/10.1006/mpev.1999.0688

Mannen, H., Nagata, Y., & Tsuji, S. (2001). Mitochondrial DNA reveal that domestic goat (Capra hircus) are genetically affected by two subspecies of bezoar (Capra aegagurus). Biochemical Genetics, 39(5–6), 145–154. https://doi.org/10.1023/A:1010266207735

Márquez, F. J., Granados, J. E., Caruz, A., Soriguer, R. C., Fandos, P., Cano-Manuel, F. J., & Pérez, J. M. (2020). Genetic diversity of cytochrome b in Iberian ibex from Andalusia. Mammalian Biology, 100(6), 675–684. https://doi.org/10.1007/s42991-020-00077-z

Mezzelani, A., Zhang, Y., Redaelli, L., Castiglioni, B., Leone, P., Williams, J. L., Toldo, S. S., Wigger, G., Fries, R., & Ferretti, L. (1995). Chromosomal localization and molecular characterization of 53 cosmid-derived bovine microsatellites. Mammalian Genome, 6(9), 629–635. https://doi.org/10.1007/BF00352370

Nomura, K., Yonezawa, T., Mano, S., Kawakami, S., Shedlock, A. M., Hasegawa, M., & Amano, T. (2013). Domestication Process of the Goat Revealed by an Analysis of the Nearly Complete Mitochondrial Protein-Encoding Genes. PLoS ONE, 8(8), e67775. https://doi.org/10.1371/journal.pone.0067775

Paterson, K. A., & Crawford, A. M. (2000). Ovine microsatellite OarKP6 isolated from a BAC containing the ovine interferon gamma gene. Animal Genetics, 31(5), 343. https://doi.org/10.1046/j.1365-2052.2000.00667.x

Pidancier, N., Jordan, S., Luikart, G., & Taberlet, P. (2006). Evolutionary history of the genus Capra (Mammalia, Artiodactyla): Discordance between mitochondrial DNA and Y-chromosome phylogenies. Molecular Phylogenetics and Evolution, 40(3), 739–749. https://doi.org/10.1016/j.ympev.2006.04.002

Sultana, S., Mannen, H., & Tsuji, S. (2003). Mitochondrial DNA diversity of Pakistani goats. Animal Genetics, 34(6), 417–421. https://doi.org/10.1046/j.0268-9146.2003.01040.x

Swarbrick, P. A., Buchanan, F. C., & Crawford, A. M. (1991). Ovine dinucleotide repeat polymorphism at the MAF36 locus. Animal Genetics, 22(4), 377–378. https://doi.org/10.1111/j.1365-2052.1991.tb00696.x

Takada, T., Kikkawa, Y., Yonekawa, H., Kawakami, S., & Amano, T. (1997). Bezoar (Capra aegagrus) is a matriarchal candidate for ancestor of domestic goat (Capra hircus): Evidence from the mitochondrial DNA diversity. Biochemical Genetics, 35(9–10), 315–326. https://doi.org/10.1023/A:1021869704889

Toldo, S. S., Fries, R., Steffen, P., Neiberg, H. L., Barendse, W., Womack, J. E., Hetzel, D. J. S., & Stranzinger, G. (1993). Physically mapped, cosmid-derived microsatellite markers as anchor loci on bovine chromosomes. Mammalian Genome, 4(12), 720–727. https://doi.org/10.1007/BF00357796

Ureña, I., Ersmark, E., Samaniego, J. A., Galindo-Pellicena, M. A., Crégut-Bonnoure, E., Bolívar, H., Gómez-Olivencia, A., Rios-Garaizar, J., Garate, D., Dalén, L., Arsuaga, J. L., & Valdiosera, C. E. (2018). Unraveling the genetic history of the European wild goats. Quaternary Science Reviews, 185, 189–198. https://doi.org/10.1016/j.quascirev.2018.01.017

**Supporting Material 3.** **Posterior probability of assignment of Iberian ibexes (*Capra pyrenaica*) and domestic goats (*Capra hircus*) to each of the two genetic clusters (Cluster 1 and Cluster 2) inferred using STRUCTURE, in the assessment of hybridization among wild and domestic goats.**

| Sample | Species | % attribution to Cluster 1 | % attribution to Cluster 2 |
| --- | --- | --- | --- |
| 16082 | *Capra pyrenaica* | 99.8 | 0.2 |
| 17070 | *Capra pyrenaica* | 99.8 | 0.2 |
| 111254 | *Capra pyrenaica* | 99.8 | 0.2 |
| 101 | *Capra pyrenaica* | 99.8 | 0.2 |
| 123 | *Capra pyrenaica* | 99.8 | 0.2 |
| 3 | *Capra pyrenaica* | 99.2 | 0.8 |
| 103 | *Capra pyrenaica* | 99.8 | 0.2 |
| 104 | *Capra pyrenaica* | 99.8 | 0.2 |
| 106 | *Capra pyrenaica* | 99.8 | 0.2 |
| 107 | *Capra pyrenaica* | 99.7 | 0.3 |
| 108 | *Capra pyrenaica* | 99.4 | 0.6 |
| 109 | *Capra pyrenaica* | 99.8 | 0.2 |
| 111 | *Capra pyrenaica* | 99.9 | 0.1 |
| 113 | *Capra pyrenaica* | 99.8 | 0.2 |
| 114 | *Capra pyrenaica* | 99.9 | 0.1 |
| 115 | *Capra pyrenaica* | 99.8 | 0.2 |
| 116 | *Capra pyrenaica* | 99.8 | 0.2 |
| 118 | *Capra pyrenaica* | 99.8 | 0.2 |
| 119 | *Capra pyrenaica* | 99.8 | 0.2 |
| 122 | *Capra pyrenaica* | 99.9 | 0.1 |
| 131 | *Capra pyrenaica* | 99.9 | 0.1 |
| 141 | *Capra pyrenaica* | 99.9 | 0.1 |
| 150 | *Capra pyrenaica* | 99.9 | 0.1 |
| 306 | *Capra pyrenaica* | 99.8 | 0.2 |
| 309 | *Capra pyrenaica* | 99.8 | 0.2 |
| 313 | *Capra pyrenaica* | 99.8 | 0.2 |
| 317 | *Capra pyrenaica* | 99.8 | 0.2 |
| 327 | *Capra pyrenaica* | 98.8 | 1.2 |
| 329 | *Capra pyrenaica* | 99.4 | 0.6 |
| 333 | *Capra pyrenaica* | 99.8 | 0.2 |
| 337 | *Capra pyrenaica* | 99.7 | 0.3 |
| 16062 | *Capra pyrenaica* | 99.8 | 0.2 |
| 16067 | *Capra pyrenaica* | 99.8 | 0.2 |
| 16068 | *Capra pyrenaica* | 99.9 | 0.1 |
| 16069 | *Capra pyrenaica* | 99.8 | 0.2 |
| 16087 | *Capra pyrenaica* | 99.8 | 0.2 |
| 16089 | *Capra pyrenaica* | 99.8 | 0.2 |
| 16090 | *Capra pyrenaica* | 99.8 | 0.2 |
| 17025 | *Capra pyrenaica* | 98.9 | 1.1 |
| 17039 | *Capra pyrenaica* | 99.7 | 0.3 |
| 17042 | *Capra pyrenaica* | 99.9 | 0.1 |
| 17058 | *Capra pyrenaica* | 99.9 | 0.1 |
| 17076 | *Capra pyrenaica* | 99.8 | 0.2 |
| 17078 | *Capra pyrenaica* | 99.8 | 0.2 |
| 17080 | *Capra pyrenaica* | 99.8 | 0.2 |
| 111234 | *Capra pyrenaica* | 99.8 | 0.2 |
| 111256 | *Capra pyrenaica* | 99.8 | 0.2 |
| 111257 | *Capra pyrenaica* | 99.8 | 0.2 |
| 19018 | *Capra pyrenaica* | 99.7 | 0.3 |
| 19019 | *Capra pyrenaica* | 99.7 | 0.3 |
| 19021 | *Capra pyrenaica* | 99.7 | 0.3 |
| 19022 | *Capra pyrenaica* | 99.8 | 0.2 |
| 19023 | *Capra pyrenaica* | 99.8 | 0.2 |
| 19024 | *Capra pyrenaica* | 99.9 | 0.1 |
| 19025 | *Capra pyrenaica* | 99.8 | 0.2 |
| 19026 | *Capra pyrenaica* | 99.8 | 0.2 |
| 19027 | *Capra pyrenaica* | 98.7 | 1.3 |
| 19028 | *Capra pyrenaica* | 99.8 | 0.2 |
| 19029 | *Capra pyrenaica* | 99.8 | 0.2 |
| 19030 | *Capra pyrenaica* | 99.8 | 0.2 |
| 19031 | *Capra pyrenaica* | 99.9 | 0.1 |
| 19032 | *Capra pyrenaica* | 99.8 | 0.2 |
| 19033 | *Capra pyrenaica* | 99.8 | 0.2 |
| 19035 | *Capra pyrenaica* | 99.8 | 0.2 |
| CP85/08 | *Capra pyrenaica* | 99.8 | 0.2 |
| MSA044 | *Capra pyrenaica* | 99.8 | 0.2 |
| MSA114 | *Capra pyrenaica* | 99.9 | 0.1 |
| 19036 | *Capra pyrenaica* | 99.9 | 0.1 |
| 19037 | *Capra pyrenaica* | 99.9 | 0.1 |
| 18092 | *Capra pyrenaica* | 99.8 | 0.2 |
| 18093 | *Capra pyrenaica* | 99.8 | 0.2 |
| 18094 | *Capra pyrenaica* | 99.5 | 0.5 |
| 18095 | *Capra pyrenaica* | 99.8 | 0.2 |
| 18096 | *Capra pyrenaica* | 99.7 | 0.3 |
| 190002 | *Capra pyrenaica* | 99.6 | 0.4 |
| 190003 | *Capra pyrenaica* | 99.3 | 0.7 |
| 19074 | *Capra pyrenaica* | 99.7 | 0.3 |
| 19075 | *Capra pyrenaica* | 99.8 | 0.2 |
| 180007 | *Capra hircus* (domestic) | 0.2 | 99.8 |
| 180010 | *Capra hircus* (domestic*)* | 0.3 | 99.7 |
| 180019 | *Capra hircus* (domestic) | 0.2 | 99.8 |
| 18021 | *Capra hircus* (domestic) | 1.2 | 98.8 |
| 18022 | *Capra hircus* (domestic) | 0.2 | 99.8 |
| 18023 | *Capra hircus* (domestic) | 0.2 | 99.8 |
| 18024 | *Capra hircus* (domestic) | 1.2 | 98.8 |
| 18025 | *Capra hircus* (domestic) | 0.2 | 99.8 |
| 18026 | *Capra hircus* (domestic) | 1.2 | 98.8 |
| 18027 | *Capra hircus* (domestic) | 0.2 | 99.8 |
| 18028 | *Capra hircus* (domestic) | 0.2 | 99.8 |
| 18029 | *Capra hircus* (domestic) | 0.2 | 99.8 |
| 18030 | *Capra hircus* (domestic) | 0.3 | 99.7 |
| 18031 | *Capra hircus* (domestic) | 0.2 | 99.8 |
| 18004 | *Capra hircus* (domestic) | 0.3 | 99.7 |
| 18006 | *Capra hircus* (domestic) | 2.1 | 97.9 |
| 180012 | *Capra hircus* (domestic) | 0.7 | 99.3 |
| 180018 | *Capra hircus* (domestic) | 0.2 | 99.8 |

**Supporting Material 5. Number of alleles and level of heterozygosity for the Iberian ibex from Cataluña assessed in this study.** (n= number of individuals; N_A_=number of alleles; Ho=Observed heterozygosity; He=Expected heterozygosity; * - monomorphic locus for that population).

|  |  | *Cataluña*  *(n=78)* | | | *Tortosa-Beseit (n=51)* | | | *Montserrat*  *(n=18)* | | | *Montgri*  *(n=9)* | | |
| --- | --- | --- | --- | --- | --- | --- | --- | --- | --- | --- | --- | --- | --- |
| *Locus* | *Locus range* | *N_A_* | *Ho* | *He* | *N_A_* | *Ho* | *He* | *N_A_* | *Ho* | *He* | *N_A_* | *Ho* | *He* |
| ILSTS29 | 151 - 177 | 6 | 0.358 | 0.516 | 4 | 0.380 | 0.468 | 4 | 0.222 | 0.347 | 3 | 0.571 | 0.615 |
| MILSTS076 | 111-147 | 8 | 0.437 | 0.419 | 7 | 0.239 | 0.259 | 4 | 0.823 | 0.543 | 3 | 0.750 | 0.658 |
| OARFCB193 | 108-150 | 5 | 0.863 | 0.733 | 5 | 0.891 | 0.750 | 4 | 0.833 | 0.663 | 3 | 0.778 | 0.569 |
| ETH10 | 189-225 | 9 | 0.547 | 0.741 | 7 | 0.627 | 0.761 | 6 | 0.615 | 0.735 | 2 | 0.000 | 0.234 |
| MAF36 | 101-121 | 7 | 0.623 | 0.631 | 4 | 0.580 | 0.534 | 3 | 0.722 | 0.541 | 4 | 0.667 | 0.732 |
| OarKP6 | 184-202 | 8 | 0.408 | 0.646 | 5 | 0.431 | 0.653 | 4 | 0.500 | 0.568 | 5 | 0.112 | 0.752 |
| BM4505 | 219-287 | 7 | 0.592 | 0.758 | 5 | 0.666 | 0.732 | 5 | 0.625 | 0.756 | 3 | 0.112 | 0.216 |
| BM1258 | 97-271 | 8 | 0.629 | 0.709 | 6 | 0.666 | 0.682 | 3 | 0.625 | 0.641 | 2 | 0.334 | 0.334 |
| SR-CRSP-8 | 193-245 | 8 | 0.522 | 0.557 | 6 | 0.533 | 0.558 | 7 | 0.687 | 0.689 | 2 | 0.125 | 0.125 |
| BM1818 | 221-283 | 4 | 0.589 | 0.675 | 3 | 0.604 | 0.671 | 4 | 0.764 | 0.668 | 2 | 0.125 | 0.525 |
| URB058 | 133-163 | 6 | 0.597 | 0.737 | 6 | 0.690 | 0.723 | 3 | 0.727 | 0.627 | 1 | * | * |
| BM1225 | 220-264 | 8 | 0.632 | 0.696 | 8 | 0.632 | 0.722 | 3 | 0.722 | 0.565 | 2 | 0.445 | 0.470 |
| IDVGA30 | 144-242 | 6 | 0.533 | 0.746 | 3 | 0.411 | 0.568 | 5 | 0.800 | 0.778 | 4 | 0.334 | 0.867 |
| JMP29 | 106-128 | 4 | 0.639 | 0.667 | 4 | 0.688 | 0.688 | 3 | 0.722 | 0.680 | 2 | 0.223 | 0.209 |
