# Supplementary material for: Genetic signature of blind reintroductions of Iberian ibex (Capra pyrenaica) in Catalonia, Northeast Spain
Source: PLoS One. 2022 Sep 21;17(9):e0269873. doi: 10.1371/journal.pone.0269873 (PMC9491545; doi:10.1371/journal.pone.0269873)
Supplement: S3 File — (DOCX) [file pone.0269873.s003.docx]

**Supporting Material 3.** **Posterior probability of assignment of Iberian ibexes (*Capra pyrenaica*) and domestic goats (*Capra hircus*) to each of the two genetic clusters (Cluster 1 and Cluster 2) inferred using STRUCTURE, in the assessment of hybridization among wild and domestic goats.**

| Sample | Species | % attribution to Cluster 1 | % attribution to Cluster 2 |
| --- | --- | --- | --- |
| 16082 | *Capra pyrenaica* | 99.8 | 0.2 |
| 17070 | *Capra pyrenaica* | 99.8 | 0.2 |
| 111254 | *Capra pyrenaica* | 99.8 | 0.2 |
| 101 | *Capra pyrenaica* | 99.8 | 0.2 |
| 123 | *Capra pyrenaica* | 99.8 | 0.2 |
| 3 | *Capra pyrenaica* | 99.2 | 0.8 |
| 103 | *Capra pyrenaica* | 99.8 | 0.2 |
| 104 | *Capra pyrenaica* | 99.8 | 0.2 |
| 106 | *Capra pyrenaica* | 99.8 | 0.2 |
| 107 | *Capra pyrenaica* | 99.7 | 0.3 |
| 108 | *Capra pyrenaica* | 99.4 | 0.6 |
| 109 | *Capra pyrenaica* | 99.8 | 0.2 |
| 111 | *Capra pyrenaica* | 99.9 | 0.1 |
| 113 | *Capra pyrenaica* | 99.8 | 0.2 |
| 114 | *Capra pyrenaica* | 99.9 | 0.1 |
| 115 | *Capra pyrenaica* | 99.8 | 0.2 |
| 116 | *Capra pyrenaica* | 99.8 | 0.2 |
| 118 | *Capra pyrenaica* | 99.8 | 0.2 |
| 119 | *Capra pyrenaica* | 99.8 | 0.2 |
| 122 | *Capra pyrenaica* | 99.9 | 0.1 |
| 131 | *Capra pyrenaica* | 99.9 | 0.1 |
| 141 | *Capra pyrenaica* | 99.9 | 0.1 |
| 150 | *Capra pyrenaica* | 99.9 | 0.1 |
| 306 | *Capra pyrenaica* | 99.8 | 0.2 |
| 309 | *Capra pyrenaica* | 99.8 | 0.2 |
| 313 | *Capra pyrenaica* | 99.8 | 0.2 |
| 317 | *Capra pyrenaica* | 99.8 | 0.2 |
| 327 | *Capra pyrenaica* | 98.8 | 1.2 |
| 329 | *Capra pyrenaica* | 99.4 | 0.6 |
| 333 | *Capra pyrenaica* | 99.8 | 0.2 |
| 337 | *Capra pyrenaica* | 99.7 | 0.3 |
| 16062 | *Capra pyrenaica* | 99.8 | 0.2 |
| 16067 | *Capra pyrenaica* | 99.8 | 0.2 |
| 16068 | *Capra pyrenaica* | 99.9 | 0.1 |
| 16069 | *Capra pyrenaica* | 99.8 | 0.2 |
| 16087 | *Capra pyrenaica* | 99.8 | 0.2 |
| 16089 | *Capra pyrenaica* | 99.8 | 0.2 |
| 16090 | *Capra pyrenaica* | 99.8 | 0.2 |
| 17025 | *Capra pyrenaica* | 98.9 | 1.1 |
| 17039 | *Capra pyrenaica* | 99.7 | 0.3 |
| 17042 | *Capra pyrenaica* | 99.9 | 0.1 |
| 17058 | *Capra pyrenaica* | 99.9 | 0.1 |
| 17076 | *Capra pyrenaica* | 99.8 | 0.2 |
| 17078 | *Capra pyrenaica* | 99.8 | 0.2 |
| 17080 | *Capra pyrenaica* | 99.8 | 0.2 |
| 111234 | *Capra pyrenaica* | 99.8 | 0.2 |
| 111256 | *Capra pyrenaica* | 99.8 | 0.2 |
| 111257 | *Capra pyrenaica* | 99.8 | 0.2 |
| 19018 | *Capra pyrenaica* | 99.7 | 0.3 |
| 19019 | *Capra pyrenaica* | 99.7 | 0.3 |
| 19021 | *Capra pyrenaica* | 99.7 | 0.3 |
| 19022 | *Capra pyrenaica* | 99.8 | 0.2 |
| 19023 | *Capra pyrenaica* | 99.8 | 0.2 |
| 19024 | *Capra pyrenaica* | 99.9 | 0.1 |
| 19025 | *Capra pyrenaica* | 99.8 | 0.2 |
| 19026 | *Capra pyrenaica* | 99.8 | 0.2 |
| 19027 | *Capra pyrenaica* | 98.7 | 1.3 |
| 19028 | *Capra pyrenaica* | 99.8 | 0.2 |
| 19029 | *Capra pyrenaica* | 99.8 | 0.2 |
| 19030 | *Capra pyrenaica* | 99.8 | 0.2 |
| 19031 | *Capra pyrenaica* | 99.9 | 0.1 |
| 19032 | *Capra pyrenaica* | 99.8 | 0.2 |
| 19033 | *Capra pyrenaica* | 99.8 | 0.2 |
| 19035 | *Capra pyrenaica* | 99.8 | 0.2 |
| CP85/08 | *Capra pyrenaica* | 99.8 | 0.2 |
| MSA044 | *Capra pyrenaica* | 99.8 | 0.2 |
| MSA114 | *Capra pyrenaica* | 99.9 | 0.1 |
| 19036 | *Capra pyrenaica* | 99.9 | 0.1 |
| 19037 | *Capra pyrenaica* | 99.9 | 0.1 |
| 18092 | *Capra pyrenaica* | 99.8 | 0.2 |
| 18093 | *Capra pyrenaica* | 99.8 | 0.2 |
| 18094 | *Capra pyrenaica* | 99.5 | 0.5 |
| 18095 | *Capra pyrenaica* | 99.8 | 0.2 |
| 18096 | *Capra pyrenaica* | 99.7 | 0.3 |
| 190002 | *Capra pyrenaica* | 99.6 | 0.4 |
| 190003 | *Capra pyrenaica* | 99.3 | 0.7 |
| 19074 | *Capra pyrenaica* | 99.7 | 0.3 |
| 19075 | *Capra pyrenaica* | 99.8 | 0.2 |
| 180007 | *Capra hircus* (domestic) | 0.2 | 99.8 |
| 180010 | *Capra hircus* (domestic*)* | 0.3 | 99.7 |
| 180019 | *Capra hircus* (domestic) | 0.2 | 99.8 |
| 18021 | *Capra hircus* (domestic) | 1.2 | 98.8 |
| 18022 | *Capra hircus* (domestic) | 0.2 | 99.8 |
| 18023 | *Capra hircus* (domestic) | 0.2 | 99.8 |
| 18024 | *Capra hircus* (domestic) | 1.2 | 98.8 |
| 18025 | *Capra hircus* (domestic) | 0.2 | 99.8 |
| 18026 | *Capra hircus* (domestic) | 1.2 | 98.8 |
| 18027 | *Capra hircus* (domestic) | 0.2 | 99.8 |
| 18028 | *Capra hircus* (domestic) | 0.2 | 99.8 |
| 18029 | *Capra hircus* (domestic) | 0.2 | 99.8 |
| 18030 | *Capra hircus* (domestic) | 0.3 | 99.7 |
| 18031 | *Capra hircus* (domestic) | 0.2 | 99.8 |
| 18004 | *Capra hircus* (domestic) | 0.3 | 99.7 |
| 18006 | *Capra hircus* (domestic) | 2.1 | 97.9 |
| 180012 | *Capra hircus* (domestic) | 0.7 | 99.3 |
| 180018 | *Capra hircus* (domestic) | 0.2 | 99.8 |

**Supporting Material 5. Number of alleles and level of heterozygosity for the Iberian ibex from Cataluña assessed in this study.** (n= number of individuals; N_A_=number of alleles; Ho=Observed heterozygosity; He=Expected heterozygosity; * - monomorphic locus for that population).

|  |  | *Cataluña*  *(n=78)* | | | *Tortosa-Beseit (n=51)* | | | *Montserrat*  *(n=18)* | | | *Montgri*  *(n=9)* | | |
| --- | --- | --- | --- | --- | --- | --- | --- | --- | --- | --- | --- | --- | --- |
| *Locus* | *Locus range* | *N_A_* | *Ho* | *He* | *N_A_* | *Ho* | *He* | *N_A_* | *Ho* | *He* | *N_A_* | *Ho* | *He* |
| ILSTS29 | 151 - 177 | 6 | 0.358 | 0.516 | 4 | 0.380 | 0.468 | 4 | 0.222 | 0.347 | 3 | 0.571 | 0.615 |
| MILSTS076 | 111-147 | 8 | 0.437 | 0.419 | 7 | 0.239 | 0.259 | 4 | 0.823 | 0.543 | 3 | 0.750 | 0.658 |
| OARFCB193 | 108-150 | 5 | 0.863 | 0.733 | 5 | 0.891 | 0.750 | 4 | 0.833 | 0.663 | 3 | 0.778 | 0.569 |
| ETH10 | 189-225 | 9 | 0.547 | 0.741 | 7 | 0.627 | 0.761 | 6 | 0.615 | 0.735 | 2 | 0.000 | 0.234 |
| MAF36 | 101-121 | 7 | 0.623 | 0.631 | 4 | 0.580 | 0.534 | 3 | 0.722 | 0.541 | 4 | 0.667 | 0.732 |
| OarKP6 | 184-202 | 8 | 0.408 | 0.646 | 5 | 0.431 | 0.653 | 4 | 0.500 | 0.568 | 5 | 0.112 | 0.752 |
| BM4505 | 219-287 | 7 | 0.592 | 0.758 | 5 | 0.666 | 0.732 | 5 | 0.625 | 0.756 | 3 | 0.112 | 0.216 |
| BM1258 | 97-271 | 8 | 0.629 | 0.709 | 6 | 0.666 | 0.682 | 3 | 0.625 | 0.641 | 2 | 0.334 | 0.334 |
| SR-CRSP-8 | 193-245 | 8 | 0.522 | 0.557 | 6 | 0.533 | 0.558 | 7 | 0.687 | 0.689 | 2 | 0.125 | 0.125 |
| BM1818 | 221-283 | 4 | 0.589 | 0.675 | 3 | 0.604 | 0.671 | 4 | 0.764 | 0.668 | 2 | 0.125 | 0.525 |
| URB058 | 133-163 | 6 | 0.597 | 0.737 | 6 | 0.690 | 0.723 | 3 | 0.727 | 0.627 | 1 | * | * |
| BM1225 | 220-264 | 8 | 0.632 | 0.696 | 8 | 0.632 | 0.722 | 3 | 0.722 | 0.565 | 2 | 0.445 | 0.470 |
| IDVGA30 | 144-242 | 6 | 0.533 | 0.746 | 3 | 0.411 | 0.568 | 5 | 0.800 | 0.778 | 4 | 0.334 | 0.867 |
| JMP29 | 106-128 | 4 | 0.639 | 0.667 | 4 | 0.688 | 0.688 | 3 | 0.722 | 0.680 | 2 | 0.223 | 0.209 |
